# Risk Factors of Cytomegalovirus Reactivation in Ulcerative Colitis Patients: A Meta-Analysis

**DOI:** 10.3390/diagnostics11111952

**Published:** 2021-10-21

**Authors:** Yafei Qin, Grace Wang, Dejun Kong, Guangming Li, Hongda Wang, Hong Qin, Hao Wang

**Affiliations:** 1 Department of General Surgery, Tianjin Medical University General Hospital, 154 Anshan Road, Heping District, Tianjin 300052, China; qinyafei92@tmu.edu.cn (Y.Q.); DejunK@tmu.edu.cn (D.K.); liguang1118@163.com (G.L.); wanghd0326@163.com (H.W.); hongqin.hust@foxmail.com (H.Q.); 2 Tianjin General Surgery Institute, Tianjin Medical University General Hospital, 154 Anshan Road, Heping District, Tianjin 300052, China; 3 Faculty of Medicine, University of Toronto, Toronto, ON M5S2E8, Canada; gracewangca@hotmail.com

**Keywords:** ulcerative colitis, cytomegalovirus, risk factors, meta-analysis

## Abstract

Cytomegalovirus (CMV) infection is associated with exacerbation of disease activity in patients with ulcerative colitis (UC). However, the risk factors for CMV reactivation in this population remain debatable. This meta-analysis was performed to identify the risk factors for CMV reactivation in UC patients. PubMed, Cochrane Library, EMBASE, Web of Science, and China National Knowledge Infrastructure were searched from the inception of these databases to 31 August 2021, with the aim of identifying studies that investigated the risk factors of CMV reactivation in UC patients. A quality assessment of the included studies was performed with the Newcastle-Ottawa Scale. The publication bias was assessed respectively via a funnel plot and Egger’s regression asymmetry test. The robustness and reliability of each outcome were evaluated by sensitivity analysis. Twenty studies were included in the final meta-analysis, comprising a total of 2099 patients with UC. A significantly higher risk of CMV reactivation was observed in patients with severe UC (OR = 1.465, 95% CI: 1.107 to 1.939, *p* = 0.008), pancolitis (OR = 2.108, 95% CI: 1.586 to 2.800, *p* = 0.0001), older age of UC onset (MD = 6.212, 95% CI: 2.552 to 9.971, *p* = 0.001), as well as use of glucocorticoids (OR = 4.175, 95% CI: 3.076 to 5.666, *p* = 0.001), immunosuppressants (OR = 1.795, 95% CI: 1.289 to 2.501, *p* = 0.001), and azathioprine (OR = 1.444, 95% CI: 1.012 to 2.061, *p* = 0.043). However, infliximab treatment was observed not to increase the occurrence of CMV reactivation in patients who suffered from UC. In contrast, 5-aminosalicylic acid (OR = 0.674, 95% CI: 0.492 to 0.924, *p* = 0.014) was associated with a lower risk of CMV reactivation. Patients with UC should be closely monitored for risk factors of CMV reactivation in order to provide timely diagnosis and antiviral treatment.

## 1. Introduction

The prevalence of ulcerative colitis (UC) has steadily increased over the last few decades [1], particularly in Europe, Canada, and the United States [2,3]. The etiology of UC is linked to a dysregulated immune response to normal mucosal resident microflora, genetic susceptibility, and infection, among other influences [4,5]. However, the accumulating literature indicates that human cytomegalovirus (CMV) raises the risk of colectomy and mortality in patients with UC [6]. CMV, which belongs to the Herpesviridae family, is an opportunistic virus with latent and reactivated characteristics [7]. CMV can remain quiescent inside a human host, but CMV replication is further activated in circumstances of immune imbalance [8]. Therefore, patients with UC are more susceptible to CMV reactivation due to a compromised intestinal immunological barrier, as well as exposure to numerous immunosuppressive agents [9,10]. The risk of reactivation of CMV has been reported to range from 21% to 34% in patients with severe UC and from 32% to 36% in patients with the steroid-refractory disease [11,12]. It is generally believed that CMV reactivation is a poor prognostic indicator among patients with UC flares [13]. This can be compounded by the inflammatory reaction and exacerbated colonic damage associated with CMV reactivation [14]. Notably, the risk of colectomy in UC patients with steroid-refractory disease appears to be lower in patients on antiviral therapy [15]. The European Crohn’s and Colitis Organization guidelines suggest prompt antiviral therapy when acute severe colitis patients have CMV reactivation [16,17]. However, it is still controversial whether the extent and severity of the UC, as well as the application of immunosuppressive agents, are related to CMV reactivation [6,12]. This study aimed to conduct a meta-analysis to evaluate the risk factors for CMV reactivation in UC patients.

## 2. Materials and Methods

### 2.1. Literature and Search Strategy

A comprehensive literature search of electronic databases, including PubMed, Cochrane Library, EMBASE, Web of Science, and China National Knowledge Infrastructure, was performed, from the inception of these databases to 31 August 2021. We retrieved studies assessing CMV reactivation in UC using the following keywords in accordance with Boolean logic: (“ulcerative colitis” OR “UC” OR “inflammatory bowel disease” OR “IBD” OR “colitis gravis” OR “ulcerative colitis type” OR “idiopathic proctocolitis”) and (“cytomegalovirus” OR “CMV” OR “salivary gland virus” OR “herpesvirus 5” OR “HHV 5”). Beyond this, references from the included articles were also manually searched to identify other potential qualifying studies that may have been missed by the database searches.

### 2.2. Inclusion and Exclusion Criteria

The articles were included in this meta-analysis as long as they met the criteria of PICOS: (I) Population: Patients with a definitive diagnosis of UC; (II) Intervention: Different disease features and therapeutic options; (III) Comparison: UC patients with or without CMV reactivation (diagnostic criteria: Intestinal tissue H&E staining reveals CMV inclusion bodies; CMV-DNA positive in intestinal tissue; CMV-DNA or pp65 positive in blood with typical deep intestinal ulcers); (IV) Outcome measures: UC severity, pancolitis, glucocorticoid, immunosuppressants, azathioprine, infliximab, 5-aminosalicylic acid (5-ASA), and age of onset; (V) Study design: An official published case–control study, cohort study, or randomized controlled trial (RCT). Exclusion criteria: (I) Diagnosis of CMV reactivation only based on the positive serological antibody or endoscopic performance, but the serological test is negative; (II) abstract, letter, editorial, expert opinion, or case report; (III) non-comparative study; (IV) study design not rigorous; (V) inadequate raw data.

### 2.3. Data Extraction and Outcome Measures

Two of the reviewers (Y.Q. and D.K.) independently extracted data from the included studies. The following essential information was captured: First, author name, year of publication, sample size, study design, outcomes, and other relevant data. The data collection and refinement statistics were summarized. Where there were conflicts of opinion, the final decision was made by another authority author (H.W.). The outcome measurements were UC severity, pancolitis, age of onset, glucocorticoid, immunosuppressants, azathioprine, infliximab, and 5-ASA.

### 2.4. Quality Assessment and Statistical Analysis

The methodological quality of the included case–control and cohort studies was evaluated using the Newcastle-Ottawa Scale (NOS) [18]. The literature quality evaluation was conducted separately by two reviewers (G.L. and H.Q.). A consensus was reached through consultation for divergence. We used Stata version 15.1 (Stata Corporation, College Station, TX, USA) for statistical analyses. When I^2^ > 50%, the data were deemed to have apparent heterogeneity. We conducted a meta-analysis using a random-effect model according to the Cochrane Handbook for Systematic Reviews of Interventions (version 5.1.0). Otherwise, a fixed-effect model was conducted. For continuous variables (age of onset), weighted mean difference (WMD) was expressed for assessment. Odds ratio (OR) was applied for the assessment of categorical variables (UC severity, pancolitis, glucocorticoid, immunosuppressants, azathioprine, infliximab, and 5-ASA).

### 2.5. Publication Bias

Publication bias was assessed respectively via a funnel plot and calculation of Egger’s regression asymmetry test [19].

### 2.6. Sensitivity Analysis

The robustness and reliability of each outcome were evaluated by sensitivity analysis. Sensitivity analysis of the effects was carried out by omitting each trial in turn and recalculating the pooled effect size [20].

## 3. Results

### 3.1. Search Results

**Figure 1:**
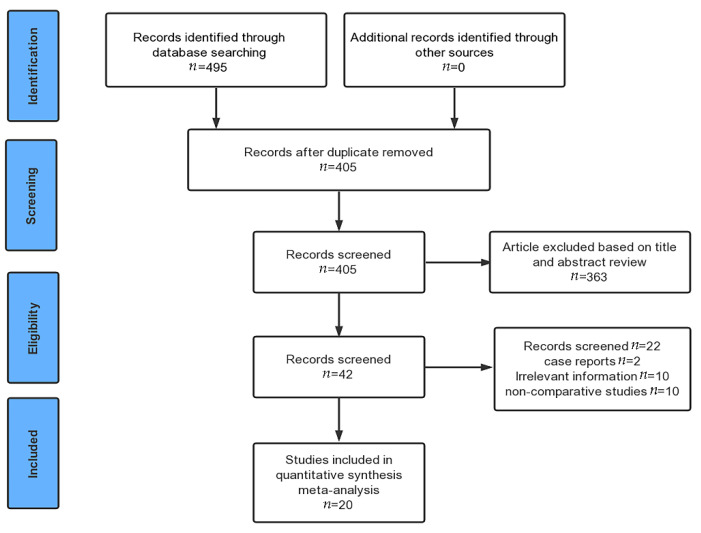
Flow chart illustrating the selection process for the articles included in the present meta-analysis.

A total of 495 studies were identified on the initial database search. We collected 405 articles after removing duplicates. By screening the abstract, 363 studies were omitted. Another 22 articles were further excluded after reading the full text. Ultimately, 20 publications [6,8,9,12,21,22,23,24,25,26,27,28,29,30,31,32,33,34,35,36] were eligible for data extraction and meta-analysis (Figure 1).

### 3.2. Characteristics of the Included Studies

The characteristics of the 20 included studies are summarized in Table 1. The included studies comprised 2099 UC patients who were incorporated into this analysis; of these, 591 patients had CMV reactivation and 1508 patients were CMV negative. 

### 3.3. Study Quality and Risk of Bias

The NOS results suggested that all eligible studies in this meta-analysis were of high quality, because their scores were equal to or greater than seven points. The details of the study quality assessments are presented in Table 2.

### 3.4. Publication Bias

**Figure 2:**
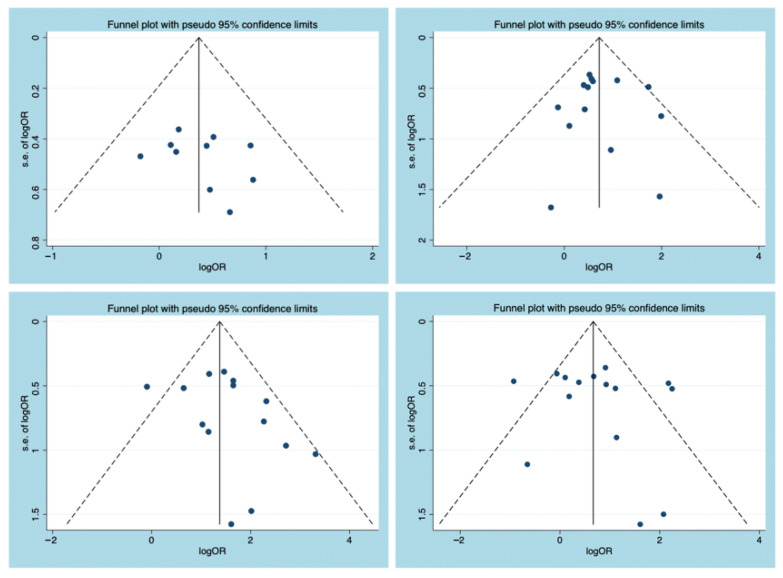
Funnel plot analysis for the publication bias assessment: (**A**) Severe UC; (**B**) pancolitis; (**C**) glucocorticoids; (**D**) immunosuppressants. UC = ulcerative colitis; OR = odds ratio; s.e. = standard error.

**Figure 3:**
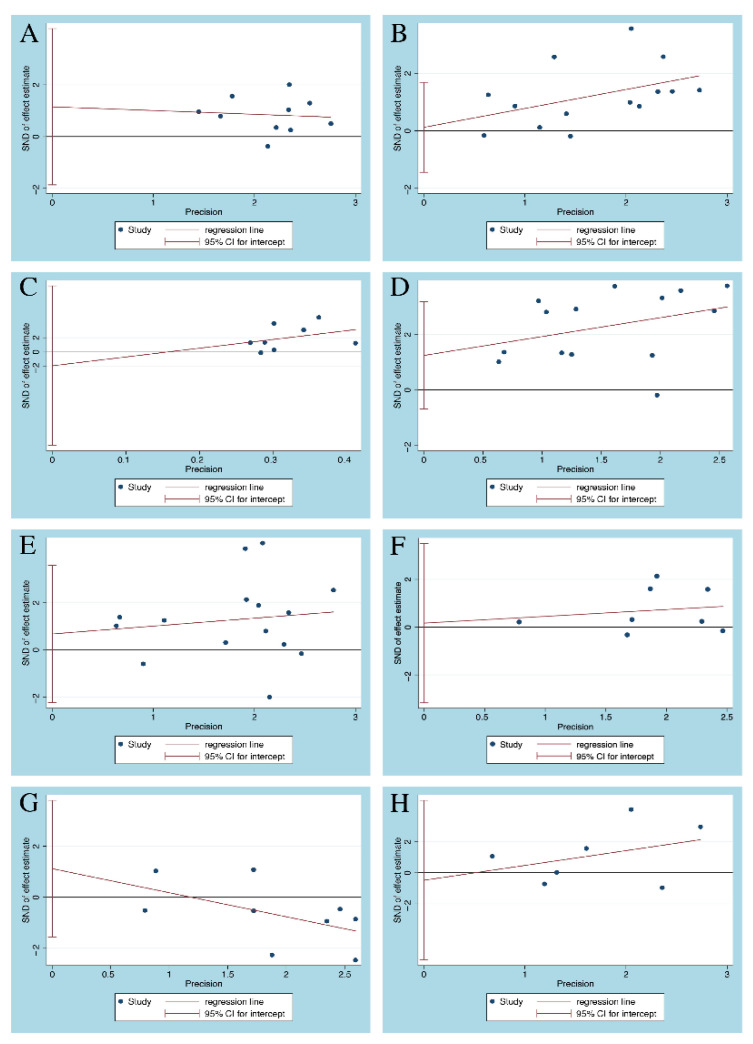
Egger’s publication bias plot: (**A**) Severe UC; (**B**) pancolitis; (**C**) age of onset; (**D**) glucocorticoids; (**E**) immunosuppressants; (**F**) azathioprine; (**G**) 5-ASA; (**H**) infliximab. UC = ulcerative colitis; 5-ASA = 5-aminosalicylic acid; SND = standard normal deviation; CI = confidence interval.

For outcomes (severe UC, pancolitis, glucocorticoid, and immunosuppressants) that were reported in more than 10 articles, Figure 2 indicates that the funnel plot was symmetrical. Meanwhile, Egger’s tests were used to statistically assess the funnel plot symmetry for all outcomes. The Egger’s tests did not indicate the presence of publication bias (Figure 3), and the specific outcomes are shown in Table 3.

### 3.5. Outcomes of the Meta-Analysis

#### 3.5.1. Severe UC

**Figure 4:**
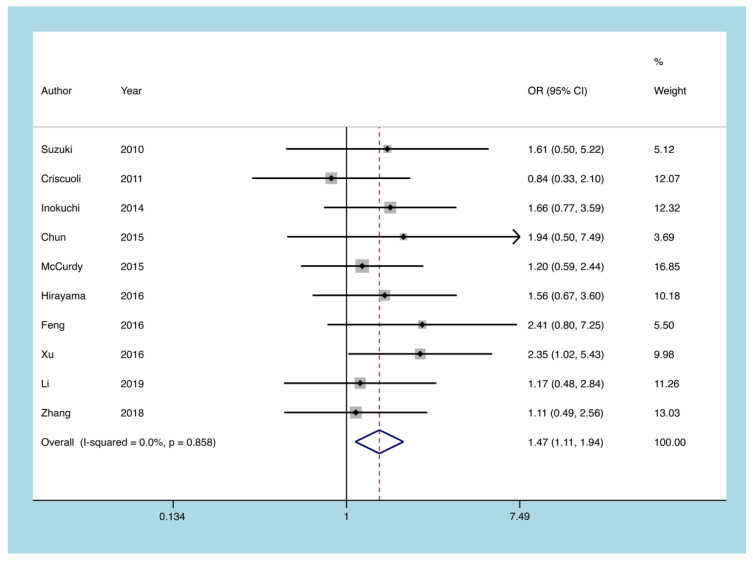
A forest plot diagram showing severe UC. UC = ulcerative colitis; OR = odds ratio.

All UC patients were evaluated by the modified Mayo scoring system and modified Truelove and Witts scoring system. The number of patients with mild-to-moderate and severe UC was extracted from ten articles. The findings indicate that the risk of CMV reactivation in severe UC was 1.465 times higher than that in mild-to-moderate UC (heterogeneity I^2^ = 0.0%, *p* = 0.858, OR = 1.465, 95% CI: 1.107 to 1.939, *p* = 0.008; Figure 4).

#### 3.5.2. Pancolitis

**Figure 5:**
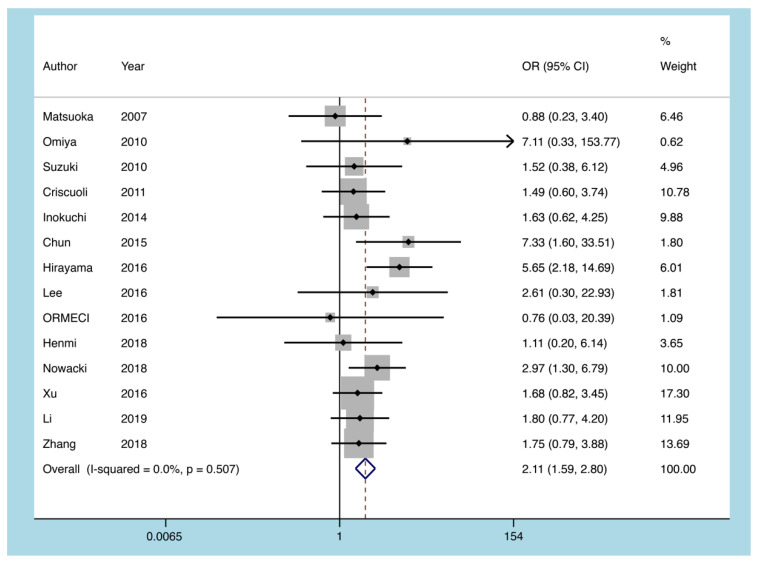
A forest plot diagram showing pancolitis. OR = odds ratio.

Fourteen articles documented colonoscopy results in patients with UC to determine the extent of intestinal lesions. There was no significant heterogeneity in the statistical results of the pooled literature (I^2^ = 0.0%, *p* = 0.507). The results of the fixed-effect model showed that the existence of pancolitis could increase the probability of CMV reactivation (OR = 2.108, 95% CI: 1.586 to 2.800, *p* = 0.0001; Figure 5).

#### 3.5.3. Age of Onset

**Figure 6:**
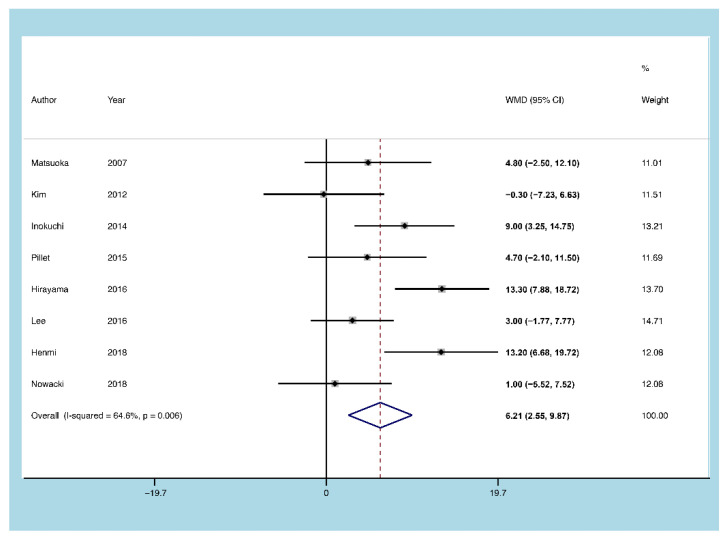
A forest plot diagram showing the age of onset. WMD = weighted mean difference.

Eight publications focused on the age of UC patients with CMV reactivation. The heterogeneity test of the included studies demonstrated substantial heterogeneity (I^2^ = 64.6%, *p* = 0.006). The results of the random-effect model showed that older age of onset of UC was associated with a higher likelihood of CMV reactivation (MD = 6.212, 95% CI: 2.552 to 9.971, *p* = 0.001; Figure 6).

#### 3.5.4. Glucocorticoids

**Figure 7:**
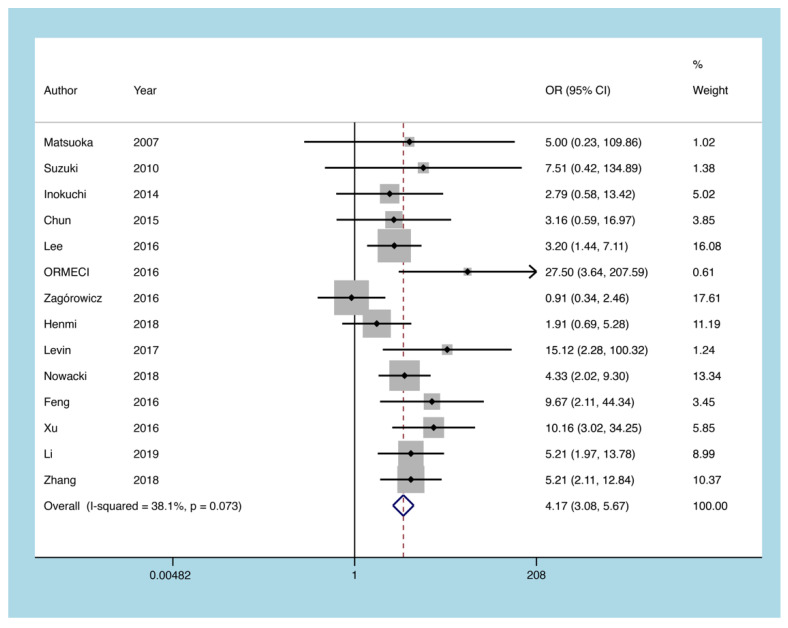
A forest plot diagram showing glucocorticoids. OR = odds ratio.

Fourteen articles described the possibility of CMV reactivation in UC patients treated with glucocorticoids. The heterogeneity test indicated that there was no heterogeneity (I^2^ = 38.1%, *p* = 0.073). The results of the fixed analysis indicated that UC patients on glucocorticoids were 4.175 times more likely to experience CMV reactivation compared to those without glucocorticoid therapy (OR = 4.175, 95% CI: 3.076 to 5.666, *p* = 0.001; Figure 7).

#### 3.5.5. Immunosuppressants

**Figure 8:**
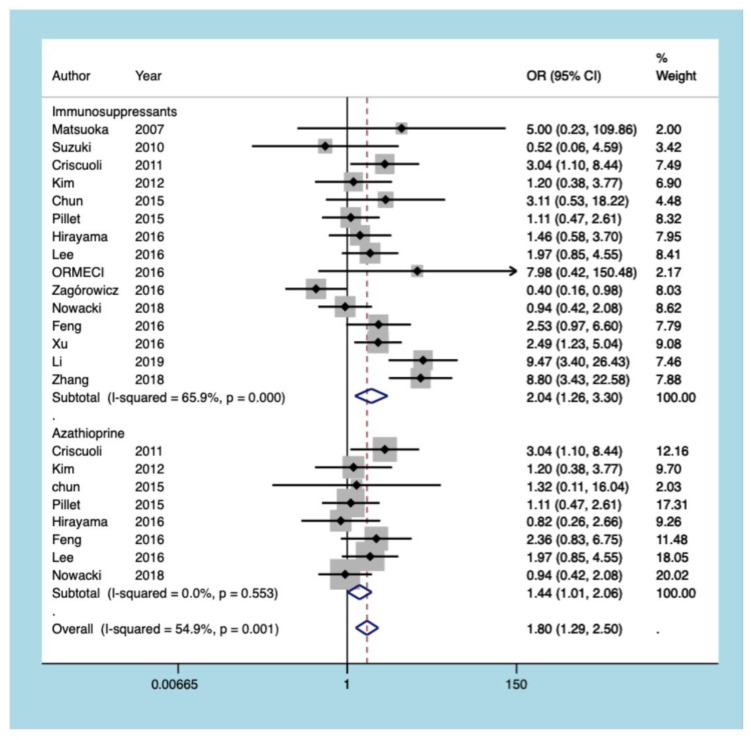
A forest plot diagram showing immunosuppressants and azathioprine. OR = odds ratio.

Fifteen articles identified the risk of CMV reactivation following the use of immunosuppressive agents (azathioprine, cyclosporine, methotrexate, 6-mercaptopurine, and mesalamine) in UC patients. The pooled results of the present meta-analysis revealed that the use of immunosuppressive agents increases the risk of CMV reactivation in patients with UC (OR = 1.795, 95% CI: 1.289 to 2.501, *p* = 0.001; Figure 8). Similarly, the results of the subgroup analysis demonstrated that azathioprine use was a risk factor for CMV reactivation (OR = 1.444, 95% CI: 1.012 to 2.061, *p* = 0.043; Figure 8).

#### 3.5.6. 5-ASA

**Figure 9:**
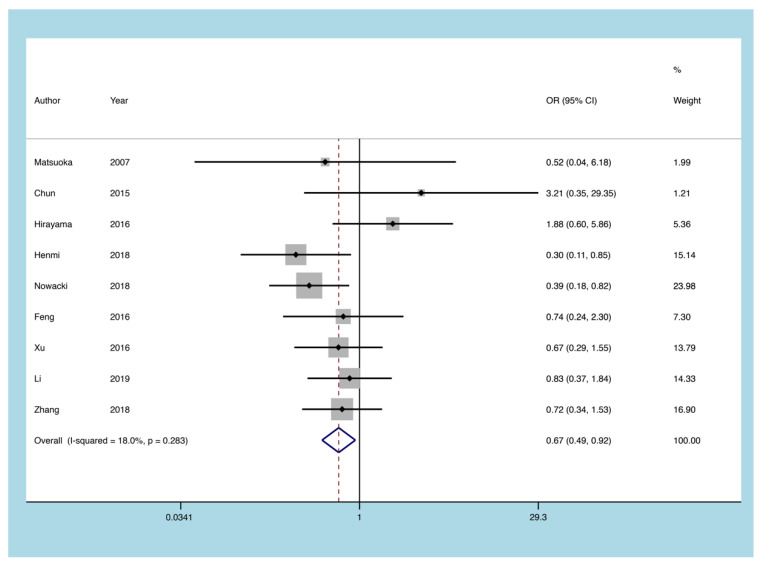
A forest plot diagram showing 5-ASA. 5-ASA = 5-aminosalicylic acid; OR = odds ratio.

Nine articles described the risk of CMV reactivation in UC patients treated with 5-ASA. The effects of the fixed-effect model revealed that 5-ASA was associated with a lower probability of CMV reactivation (heterogeneity I^2^ = 18.0%, *p* = 0.283, OR = 0.674, 95% CI: 0.492 to 0.924, *p* = 0.014; Figure 9).

#### 3.5.7. Infliximab

**Figure 10:**
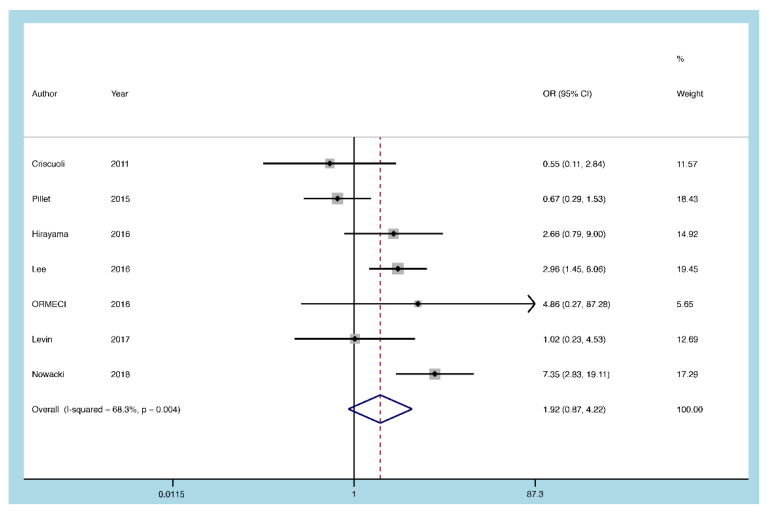
A forest plot diagram showing infliximab. OR = odds ratio.

Seven articles assessed whether infliximab affected CMV infection or reactivation in UC patients. There was no obvious heterogeneity (I^2^ = 68.3%, *p* = 0.004). A random-effect model was used. Pooling the results demonstrated that infliximab therapy was not a risk factor for CMV reactivation (OR = 1.915, 95% CI: 0.870 to 4.217, *p* = 0.107; Figure 10).

#### 3.5.8. Sensitivity Analysis

**Figure 11:**
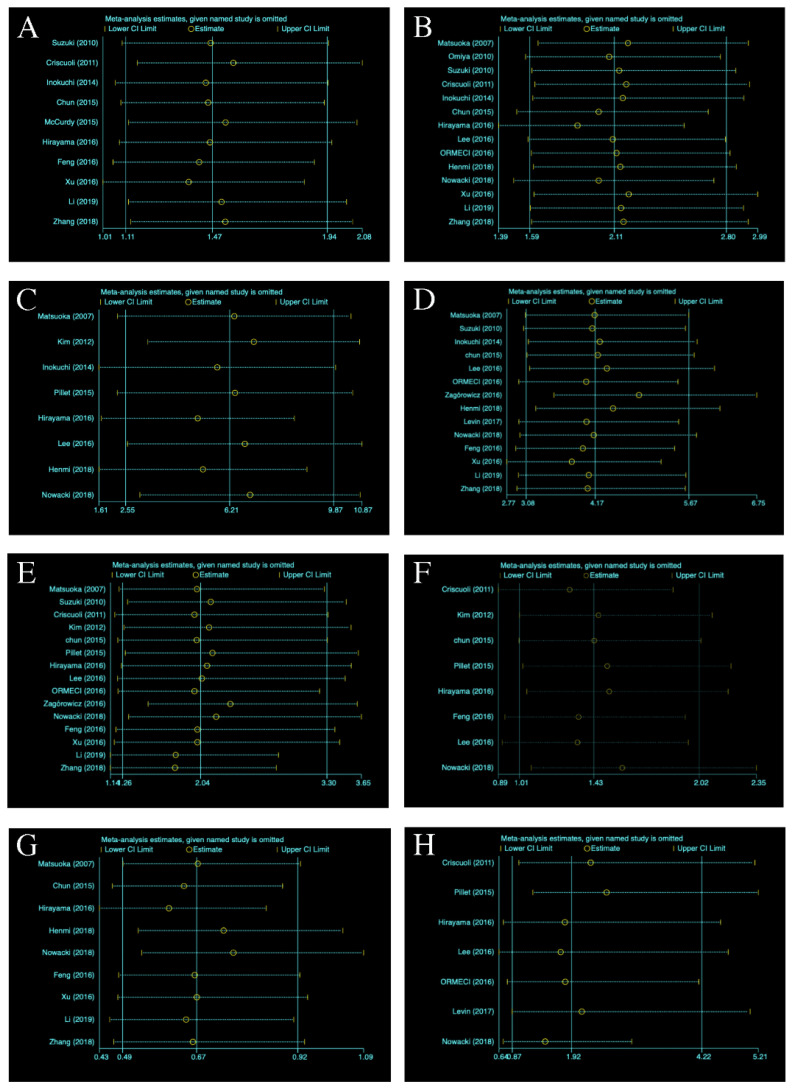
Sensitivity analysis of all outcomes: (**A**) Severe UC; (**B**) pancolitis; (**C**) age of onset; (**D**) glucocorticoids; (**E**) immunosuppressants; (**F**) azathioprine; (**G**) 5-ASA; (**H**) infliximab. UC = ulcerative colitis; 5-ASA = 5-aminosalicylic acid; CI = confidence interval.

As shown in Figure 11, the results of the sensitivity analysis demonstrated that the effects of all the risk factors on CMV reactivation in UC patients remained consistent after removing the trials one by one.

## 4. Discussion

The relationship between CMV reactivation and UC has been reviewed for several decades. Several studies have shown that CMV reactivation carries an increased risk of surgical complications and mortality in patients with UC [37]. A large number of proinflammatory cytokines such as TNF-α, IFN-γ, and IL-2 could activate the expression of transcription factor activator protein-1 (AP-1) and nuclear factor kappa B (NF-κB), thereby increasing the expression of MIP-1, CCL5, MCP-1, and adhesion molecules (ICAM-1 and VCAM-1) involved in inflammation [38,39]. Stimulated by these cytokines, monocytes, and dendritic cells phagocytosing CMV travel to the intestinal mucosal injury area by chemotaxis. TNF-α and IFN-γ mediate the activation of CMV in monocytes and dendritic cells, respectively. Furthermore, Smith et al. [40] showed that the monocytes infected with latent CMV infiltrated tissues and differentiated into macrophages that specifically expressed CDl4, TLR4, and TLR5. On the one hand, macrophages, particularly CDl4^+^ macrophages, also play an essential role in “saving cells” supporting CMV from latent to active state [41,42,43]. On the other hand, CMV reactivation promotes the expression of myeloiddifferentiationfactor88 (MyD88) and the phosphorylation of IKBα and NF-κB, contributing not just to replication but also to infectivity of CMV [44]. Taken together, CMV could penetrate the lamina propria and induce an abnormal mucosal immune reaction, exacerbating the systemic inflammatory response of the intestine [45].

More severe UC was associated with a higher risk of CMV reactivation. In this study, we found that the risk of CMV reactivation in patients with severe UC was 1.465 times higher than that of patients with mild-to-moderate UC. Furthermore, the risk of CMV reactivation was 2.108 times higher in patients with pancolitis compared to those with limited left colon lesions. These findings are supported by Nowacki [27], holding that severe UC and pancolitis are predictive factors for CMV reactivation. Indeed, severe UC and pancolitis indicate more intense inflammatory reactions, substantial intestinal mucosal barrier disruptions, and increased intestinal mucosal permeability, all of which may increase CMV reactivation.

The correlation between age of UC onset and risk of CMV reactivation remains controversial. Criscuoli [8] evaluated the natural history of CMV reactivation in a series of UC patients and found that there were no significant differences in the risk of CMV reactivation between patients ≥50 vs. <50 years of age. However, Pillet et al. [10] claimed that the age of UC onset over the age of 30 years is a contributing factor for CMV reactivation. This present meta-analysis indicated that UC patients with a later age of onset are more likely to suffer from CMV reactivation. Further examination of the articles included in this review revealed that the studies that found the age of UC onset to have little impact on CMV reactivation had enrolled UC patients from a narrow age range, primarily between 42 and 46 years old [6].

In addition to the disease features of UC patients, the association between treatment strategies and CMV reactivation should be noted. Glucocorticoids in various forms (mainly including hydrocortisone, dexamethasone, prednisolone, and prednisone) are highly potent steroid hormones on the front line of UC treatment. This meta-analysis found that glucocorticoid therapy is a risk factor for CMV reactivation, raising the risk by 4.175 times. Lee et al. [6] suggested that an average glucocorticoid use of more than 40 mg per day within one month would increase the incidence of CMV reactivation. Others have concluded that a cumulative glucocorticoid usage of greater than 400 mg within four weeks is a risk factor [25]. While the relationship between glucocorticoids and CMV reactivation was well established in our study, further investigation into the precise frequency and dose of glucocorticoids is still needed. This study also found that immunosuppressive agents are a risk factor for CMV reactivation. Likely, other studies have shown that both the reactivation and proliferative abilities of CMV are augmented by immunosuppressants [37]. Azathioprine, a cost-effective and widely available immunosuppressant for UC, was determined to be a risk factor for CMV reactivation in the subgroup analysis. The combined assessment of different immunosuppressants, including azathioprine, cyclosporine, methotrexate, 6-mercaptopurine, and mesalamine, in our study contributed to heterogeneity and may have impacted our analyses. Future studies should more clearly establish the association between CMV reactivation and individual immunosuppressants used in UC.

Interestingly, we found that 5-ASA use correlated with a lower risk of CMV reactivation. This may be confounded by the fact that patients treated with 5-ASA have milder disease and a subsequently lower incidence of CMV reactivation. Infliximab, a chimeric monoclonal immunoglobulin G1, and TNF-α antagonist, has been shown to block inflammatory responses and clear CMV infections [6,12]. As we know, TNF-α facilitates the reactivation of CMV in monocytes and dendritic cells [42]. Consistent with the results of the previous study, this meta-analysis demonstrated that infliximab is not a risk factor for CMV reactivation in UC patients [27].

Although we systematically proved that severe UC, pancolitis, older age of UC onset, glucocorticoids, immunosuppressants, and azathioprine are risk factors for CMV reactivation in UC patients, this study has several limitations. First, the relationship between more biological agents such as vedolizumab should be identified. Second, further research is required to determine the role of different immunosuppressants in CMV reactivation in UC patients.

## 5. Conclusions

Severe UC, pancolitis, glucocorticoids, immunosuppressants, azathioprine, and later age of onset are risk factors for CMV reactivation in patients with UC. Patients with these disease characteristics should be proactively screened for CMV reactivation in order to facilitate early diagnosis and timely antiviral treatment.

## Figures and Tables

**Table 1:** The basic characteristics of the studies included in the meta-analysis.

Author	Year	Country	Study Design	Study Group		Gender		Age *		Disease Duration (Years)	
				CMV^+^	CMV^−^	CMV^+^ (M/F)	CMV^−^ (M/F)	CMV^+^	CMV^−^	CMV^+^	CMV^−^
Matsuoka [25]	2007	Japan	Retrospective	25	23	12/13	17/6	42 ± 14.5	37.2 ± 11.2	4.85 ± 3.85	5.35 ± 5.00
Omiya [28]	2010	Japan	Prospective	7	13	NA	NA	NA	NA	NA	NA
Suzuki [31]	2010	Japan	Retrospective	15	58	8/7	26/32	NA	NA	7.14 ± 7.41	4.12 ± 7.39
Criscuoli [8]	2011	Italy	Prospective	28	57	19/9	31/26	NA	NA	NA	NA
Kim [12]	2012	Korea	Prospective	31	41	20/11	28/13	43.1 ± 15.4	43.4 ± 14.1	2.4 ± 2.65	3.7 ± 4.94
Inokuchi [23]	2014	Japan	Retrospective	40	78	26/14	31/47	45 ± 14.2	36 ± 12.5	1.5 ± 0.60	4.6 ± 0.53
Chun [21]	2015	Korea	Retrospective	12	31	6/6	14/17	NA	NA	NA	NA
McCurdy [26]	2015	USA	Retrospective	45	139	NA	NA	NA	NA	NA	NA
Pillet [30]	2015	France	Prospective	39	70	28/11	44/26	51.2 ± 17.0	46.5 ± 18.0	6.59 ± 7.00	6.67 ± 6.75
Hirayama [22]	2016	Japan	Retrospective	34	115	19/15	64/51	42.3 ± 14.4	29.0 ± 14.4	4.6 ± 4.9	6.0 ± 7.4
Lee [6]	2016	Korea	Retrospective	50	99	30/20	50/49	45 ± 14.75	42 ± 10.33	2.2 ± 3.25	4.7 ± 4.83
ORMECI [29]	2016	Turkey	Retrospective	8	35	6/2	17/18	NA	NA	NA	NA
Zagórowicz [32]	2016	Poland	Retrospective	33	62	22/11	39/23	NA	NA	NA	NA
Henmi [9]	2018	Japan	Retrospective	26	60	15/11	38/22	55.0 ± 13.2	41.8 ± 16.2	5.95 ± 5.07	6.67 ± 7.45
Levin [24]	2017	USA	Retrospective	13	15	6/7	12/3	NA	NA	NA	NA
Nowacki [27]	2018	Germany	Retrospective	34	205	25/9	108/97	37 ± 17.8	36 ± 18.9	9.0 ± 8.4	4.4 ± 7.3
Feng [35]	2016	China	Retrospective	31	60	18/13	39/21	NA	NA	NA	NA
Xu [36]	2016	China	Retrospective	49	143	28/21	82/61	NA	NA	NA	NA
Li [34]	2019	China	Retrospective	34	91	21/13	49/42	NA	NA	3.92 ± 1.83	4.84 ± 2.31
Zhang [33]	2018	China	Retrospective	37	113	23/14	61/52	NA	NA	3.94 ± 1.95	4.86 ± 2.32

* The age of the patients when the disease was first diagnosed; CMV = cytomegalovirus; M = male; F = female; NA = not available.

**Table 2:** Quality assessment using the NOS for risk of bias of studies included in the meta-analysis.

Study	Year	Selection	Comparability	Exposure	Total
Matsuoka	2007	4	3	2	9
Omiya	2010	4	2	2	8
Suzuki	2010	4	2	3	9
Criscuoli	2011	4	2	2	8
Kim	2012	4	2	3	9
Inokuchi	2014	3	3	2	8
Chun	2015	4	2	3	9
McCurdy	2015	4	2	3	9
Pillet	2015	4	2	3	9
Hirayama	2016	3	2	3	8
Lee	2016	4	2	3	9
ORMECI	2016	4	2	2	8
Zagórowicz	2016	3	2	2	7
Henmi	2018	3	2	3	8
Levin	2017	4	2	3	9
Nowacki	2018	4	1	3	8
Feng	2016	4	1	3	8
Xu	2016	4	2	2	8
Li	2019	3	2	2	7
Zhang	2018	4	1	3	8

NOS = Newcastle-Ottawa Scale.

**Table 3:** The results of the Egger’s tests for outcomes.

	Number of Studies	Std_Eff	*t*	*p* > |*t*|	95% CI	
Severe UC	10	Slope	−0.24	0.813	−1.521	1.231
		Bias	0.88	0.404	−1.866	4.172
Pancolitis	14	Slope	1.66	0.123	−0.206	−1.442
		Bias	0.17	0.868	−1.442	1.686
Age of onset	8	Slope	0.87	0.419	−22.170	9.180
		Bias	−0.42	0.690	−12.965	9.180
Glucocorticoids	14	Slope	1.28	0.224	−0.477	1.841
		Bias	1.40	0.186	−0.688	3.184
Immunosuppressants	15	Slope	0.48	0.642	−1.186	1.854
		Bias	0.50	0.623	−2.391	3.598
Azathioprine	8	Slope	0.41	0.699	−1.430	1.998
		Bias	0.12	0.906	−3.169	3.507
5-ASA	9	Slope	−1.66	0.140	−2.285	0.399
		Bias	0.99	0.354	−1.554	3.803
Infliximab	7	Slope	0.87	0.423	−1.860	3.770
		Bias	−0.24	0.821	−5.632	4.675

UC = ulcerative colitis; 5-ASA = 5-aminosalicylic acid.
